# A statistical approach for evaluating the effectiveness of heartworm preventive drugs: what does 100% efficacy really mean?

**DOI:** 10.1186/s13071-017-2440-x

**Published:** 2017-11-09

**Authors:** Anand N. Vidyashankar, Pablo D. Jimenez Castro, Ray M. Kaplan

**Affiliations:** 1 Department of Statistics, George Mason University, Fairfax, VA USA; 2 Department of Infectious Diseases, College of Veterinary Medicine, University of Georgia, Athens, GA 30602 USA

**Keywords:** *Dirofilaria immitis*, Efficacy, Macrocyclic lactone, Canine heartworm, Parametric bootstrap, Statistical

## Abstract

**Background:**

Initial studies of heartworm preventive drugs all yielded an observed efficacy of 100% with a single dose, and based on these data the US Food and Drug Administration (FDA) required all products to meet this standard for approval. Those initial studies, however, were based on just a few strains of parasites, and therefore were not representative of the full assortment of circulating biotypes. This issue has come to light in recent years, where it has become common for studies to yield less than 100% efficacy. This has changed the landscape for the testing of new products because heartworm efficacy studies lack the statistical power to conclude that finding zero worms is different from finding a few worms.

**Methods:**

To address this issue, we developed a novel statistical model, based on a hierarchical modeling and parametric bootstrap approach that provides new insights to assess multiple sources of variability encountered in heartworm drug efficacy studies. Using the newly established metrics we performed both data simulations and analyzed actual experimental data.

**Results:**

Our results suggest that an important source of modeling variability arises from variability in the parasite establishment rate between dogs; not accounting for this can overestimate the efficacy in more than 40% of cases. We provide strong evidence that ZoeMo-2012 and JYD-34, which both were established from the same source dog, have differing levels of susceptibility to moxidectin. In addition, we provide strong evidence that the differences in efficacy seen in two published studies using the MP3 strain were not due to randomness, and thus must be biological in nature.

**Conclusion:**

Our results demonstrate how statistical modeling can improve the interpretation of data from heartworm efficacy studies by providing a means to identify the true efficacy range based on the observed data. Importantly, these new insights should help to inform regulators on how to move forward in establishing new statistically and scientifically valid requirements for efficacy in the registration of new heartworm preventative products. Furthermore, our results provide strong evidence that heartworm ‘strains’ can change their susceptibility phenotype over short periods of time, providing further evidence that a wide diversity of susceptibility phenotypes exists among naturally circulating biotypes of *D. immitis*.

## Background

Initial studies of heartworm preventive products containing drugs of the macrocyclic lactone (ML) class all yielded an observed efficacy of 100% with a single dose [1–4]. The experimental design used for these and other studies varied slightly, but the basic design was as follows. Dogs were infected with 30 to 50 infective L3 (iL3) stage larvae and then assigned randomly to a non-treated control group and one or more drug-treated groups. Group sizes varied among studies, but six to ten dogs per group were most common. Thirty days after inoculation with the iL3 larvae, the treated group(s) was administered a single dose of the drug. Necropsy and complete worm recoveries were done 5 to 7 months after infection to allow development to the adult parasite stage.

Because all early studies yielded 100% efficacy, the US Food and Drug Administration (FDA) chose to require that all drugs licensed for the prevention of heartworm infections meet the 100% efficacy standard for approval. The first heartworm preventive product, which contained ivermectin (Heartgard-30®, Merial), received regulatory approval in 1987 [5], and this requirement for 100% efficacy has continued to this day. Those initial studies, however, were based almost entirely on a single strain of *Dirofilaria immitis* (UGA-TRS) that had been isolated from a dog in the late 1960s, and then passaged in the laboratory approximately every 3 years to about the F10 generation until 2000 [6]. From the mid 1970s until the late 1990s, most heartworm preventive studies for all of the ML drugs submitted by animal health companies to the Center for Veterinary Medicine (CVM)/FDA for product approval (ivermectin, milbemycin oxime, selamectin, moxidectin) were conducted using different generations of this same strain [6]. Furthermore, it is estimated that no more than 15 (and probably less) other strains from other laboratories were ever used for those original studies, and most of those strains were used only once and never serially propagated in the laboratory [6].

Moreover, early publications demonstrating efficacy of these products consistently do not mention the name or geographic origin of the heartworm strain used in the study, nor do they mention whether the strain was cycled in the laboratory, and if so for how long. These issues were completely ignored; authors of these papers only state how many iL3 were administered. Thus it is not possible to know the exact number of strains used or anything else about them. Given this lack of documentation, it is impossible to know how many strains were used in the early studies of ML efficacy against heartworms. Nevertheless, it is clear that (a) only a small number of heartworm strains were ever tested, (b) geographic diversity and other factors that might affect the phenotypic and/or genotypic diversity of *D. immitis* biotypes circulating throughout the US were not considered, (c) none of these strains underwent any formal or standardized process of characterization, and (d) the most commonly used strain was maintained in the laboratory for more than 30 years and passaged to about the F10 generation [6].

In the above discussion, we use the term ‘strain,’ and here it is germane to discuss what is actually meant by the term. Isolates of *D. immitis* are most frequently referred to in the heartworm literature as ‘strains,’ and this nomenclature serves a purpose of convenience to distinguish one laboratory isolate from another. The term strain implies genetic uniformity, however, such as a genetic variant or subtype of an organism. Among parasitic protozoans, the term strain is usually restricted to a homogeneous population possessing a set of defined characters [7]. Given the high levels of genetic diversity typical of nematodes, and reproduction by sexual means, calling a parasitic nematode isolate a strain would rarely be scientifically accurate. However, *D. immitis* isolates are somewhat different than most other nematode species in that they undergo a fairly severe genetic bottleneck each time they are passaged, because only about 15 to 35 worms will typically establish in a dog following an inoculation of 50 iL3. In addition, helminth parasites have a subdivided population structure as adult worms because they are confined to their definitive host and are only able to mate with worms co-inhabiting the same host [8]. As a result many microfilariae circulating in the blood of an infected dog are partial or full siblings, and given that relatively few adult worms of each sex are present in a given dog, sibling matings in the F2 generation are probable, and increase in probability with each passage. This process greatly reduces genetic diversity in a heartworm ‘strain’ compared with what would exist with the establishment and passage of a strongylid gastrointestinal nematode, where thousands of worms typically establish with each passage. Furthermore, this inherent bottlenecking that occurs each time a heartworm isolate is passaged, can cause the ‘strain’ to change each time it is passaged, particularly in the first few passages. The extent of these changes likely can vary greatly from ‘strain’ to ‘strain’ depending on the level of genetic diversity present in the original field infection and the number of worms establishing each subsequent generation. The UGA-TRS strain used for the majority of product approval studies prior to 2000 [6], would be predicted to be highly bottlenecked and have very low genetic diversity as a consequence of the repeated passage of this strain over 30 years.

In part to address this issue, in 2000 the FDA-CVM changed policy to require the use of newly acquired strains from different geographic regions in the US. Then in 2006, FDA-CVM unofficially refined this policy to require that approval studies for new products use a strain that was isolated no more than 3 years earlier. These policy changes led to the isolation and maintenance of additional strains of *D. immitis*. Between 2000 and 2010, TRS Laboratories (Athens, GA) isolated seven additional strains that were used in the majority of, if not all, heartworm efficacy studies performed in the US (by independent laboratories) over that time period [6]. These strains used would have undergone varying numbers of passages after being established in the laboratory, thus the degree of genetic and phenotypic change over time and between studies likely varied for each strain. Furthermore, because any given strain can only be used for 3 years, the strains used in product testing are constantly changing. Consequently, there is a high likelihood that any new product will be tested against a strain that represents a biotype that differs in genetic background and ML susceptibility from those strains that were tested previously. Finally, the limited numbers of strains available for testing at any one time virtually assure that the strains used to test any new product will not be representative of the full assortment of naturally circulating biotypes of *D. immitis*.

### Defining efficacy and variability in efficacy – An issue that needs reexamination

*Efficacy* can be defined as a quantitative measure of the effectiveness of a drug intended to produce a desired effect. With regard to anthelmintics, the *expected* or *‘true’ efficacy* can be defined as the average efficacy of a given drug at the population level; meaning across the entire population of dogs of varying breed, age, sex, weight, body mass, health status, etc., that are infected with heartworms of differing biotypes at different infection intensities. Because one would have to test every dog in the population to determine the true efficacy, this is always an unknown value that is estimated. Alternatively, one can estimate the true efficacy fairly accurately if both numbers of infected dogs tested and numbers of worms recovered from non-treated animals are very large (thousands of dogs of various signalments infected with tens of thousands of worms). Because this is not a reasonable endeavor, we typically test a small sample of dogs that are relatively uniform with regard to breed, age, weight, etc. This yields an observed efficacy that may or may not accurately represent the true efficacy. Clinical assessments of efficacy are then typically made based on the observed efficacy of a drug in a single study, and regulatory assessments are based on observed efficacies across several studies.

An observed efficacy of 100% means there is no variability in the efficacy data of a given study. Once efficacy falls below 100%, however, variability in efficacy will always be present between animals in the same study and between studies. This will be true whether or not the same or different strains are used, although if using different strains or biotypes variability is expected to be greater. In addition, in heartworm studies, the observed efficacy depends on the establishment rate and this value is always unknown for treated dogs. Consequently, every time a test for efficacy is performed the result will be different, and the magnitude of the difference will depend on the amount of variability in the establishment rate and the response to the treatment. Thus, the efficacy of a drug in any drug trial is not a fixed number, but lies within a set of possible values. This set of values can be described using a probability distribution whose parameters have both biological and statistical meaning [9]. To illustrate this point, in one of the early studies with ivermectin, oral tablet doses of 2.0 and 3.3 μg/kg administered 30 days after infection were 100% effective in preventing development to adults, but those same doses were only 97.2% and 98.1% effective, respectively, in a second study [3]. In yet a third study, an oral tablet dose of 2.0 μg/kg yielded only 83.3% efficacy [5]. None of these publications provide any information about which strain of *D. immitis* was used, so one or more of these studies may have used a strain other than UGA-TRS. Regardless of whether these data represent testing done with a single strain, or with multiple strains, the 2.0 μg dose yielded efficacy results ranging from 83.3% to 100% illustrating the variability that is inherent in parasite efficacy studies. In the realm of heartworm preventive drugs, however, this issue has since been virtually ignored because of the expectation of 100% efficacy at label dosages. Consequently, the issue of *D. immitis* biotype/genetic diversity and the potential differences in ML susceptibility of these various biotypes were not considered by industry and regulatory authorities. Admittedly, this issue is not important if at the label dose, differences in biotype/strain susceptibility are masked by an observed efficacy of 100% against all of them. The key question, however, which was never addressed, is “how many strains must be tested before one can reasonably conclude that all circulating biotypes of *D. immitis* would demonstrate 100% susceptibility to a single label dose of ML drug, when tested using the accepted/typical experimental model?”

### A new phenotype appears

The second and third strains isolated by TRS Laboratories, 'Butch' and 'Missouri' (2000), demonstrated the same highly susceptible phenotype as the original TRS-UGA strain. In 2006, however, the MP3 strain, only the fourth strain isolated by TRS, demonstrated a different phenotype. For the first time, it was reported that a single label dose of both ivermectin and milbemycin oxime failed to achieve 100% efficacy [10]. In that study, one heartworm was found in one dog in each of the milbemycin oxime and ivermectin treatment groups. In an attempt to meet the 100% efficacy requirement, additional studies were performed, using the formulated product (containing both spinosad and milbemycin oxime) at label dosages, to see if administering two or three doses at monthly intervals would achieve 100% [11]. In those studies one treated dog had one worm in one of the 2X treated groups (99.6% efficacy), and no worms were present in the second 2X group or in the 3X treated group (Table 1). Based on these (and possibly other) data, the FDA-CVM approved that product, but it was required that the label state that the drug should be administered “once monthly for at least 3 months after exposure to mosquitoes.” It is noteworthy that the same product when tested against the Michigan strain (first isolated in 2007 by TRS Labs) yielded 100% efficacy with a single dose. Thus, a product that was just as effective as previously approved products required an additional label statement solely because it was tested by chance against a strain/biotype demonstrating a phenotype that was ‘less susceptible’ than those few strains used previously for product testing.

**Table 1:** Efficacy data for various trials reported in Snyder et al. [10] and [11] using the MP3 strain of *Dirofilaria immitis* and treatment using milbemycin oxime (0.5–0.75 mg/kg) and spinosad (30–45 mg/kg)

Treatment regimen^a,b^	Number of worms recovered in treated dogs	Geometric mean	Model-based mean	Model-based 95% CI
30 days^a^	1	99.8	99.8	99.2, 100
30 days^b^	5	98.99	98.51	97.36, 99.44
45 days^b^	5	98.87	98.52	97.37, 99.45
30 and 60 days^b^	1	99.63	99.51	98.59, 100
15 and 45 days^b^	0	100	100	N/A
30, 60 and 90 days^b^	0	100	100	N/A

One, two or three doses of drug were administered either 30, 45, 30/60, 15/45, or 30/60/90 days post inoculation with infective L3. Model based mean and 95% confidence intervals (CI) were calculated using the parametric bootstrap model described in the text

^a^Data from Snyder et al., [10]

^b^Data from Snyder et al., [11]

These data raised important questions about how efficacy of heartworm drugs is measured and interpreted. It is important to observe here that the assessments described so far are based on an observed efficacy without taking variability into account. As an alternative approach, it is preferable to analyze the data statistically, whereby variability is taken into account and the analyses provide interval estimates referred to as confidence intervals. With this information, one can determine with high probability whether the true (unknown) efficacy falls within this interval. One can then arrive at regulatory guidelines using the end points of the confidence interval.

Prior to the studies with the MP3 strain referred to above, every new ML product approval study yielded 100% efficacy, thus interpretation of the data were straightforward. Statistical analysis of efficacy data seemed unimportant, demonstrated by the fact that in many, if not most of the early publications on efficacy of heartworm preventives, statistical analyses were not performed. In many of the more recent publications statistical analyses are performed, and the most common approach is to first log transform the data, use geometric means to calculate the efficacy, and then perform analysis with a nonparametric test such as the Wilcoxon’s Rank Sum Test [12]. Geometric means are used because parasite data are usually over-dispersed and log transformation creates a less skewed distribution, and hence is less dominated by a small percentage of high values [13]. This produces a more appropriate estimate of central tendency, and normalizes data enabling the assumption of normality required for parametric analyses. In fact, VICH international harmonization guidelines for anthelmintic efficacy recommend this approach [14]. Geometric means produce a bias, however, which causes differences in intensity to be exaggerated [13]. Moreover, a primary purpose of the log transformation is to enable the assumption of normality so that a parametric analysis can be performed. This is because parametric analyses are preferred whenever possible because nonparametric tests have low power [15], yet nonparametric analyses are most common in the heartworm literature. This is not appropriate for several reasons, including the fact that the above-described methods do not take into account variability in the establishment rate. In addition, due to high cost and logistical considerations, heartworm efficacy studies tend to use relatively few dogs, and the high pathogenicity of *D. immitis* means there are relatively few worms per dog. As a consequence, heartworm efficacy studies have innate low statistical power; thus parametric analyses are recommended. Furthermore, it is worth asking why parasitologists are still using an analysis method (Wilcoxon’s Rank Sum Test) published in 1945, before the existence of computers, when new powerful analyses exist that are much more appropriate for these types of data?

Now, let us return to the data reported by Snyder et al. [10, 11], where less than 100% efficacy was demonstrated against the MP3 strain with a single dose, both 100% and less than 100% efficacy were demonstrated with two doses administered 30 days apart, and 100% efficacy was demonstrated when three doses were administered at 30-day intervals (Table 1). Examination of these data leads to an important question: are these results really biologically different from one another? Or were these different results more likely due to variability? Examination of the 95% confidence interval (CI) suggests that variability was most likely responsible for the differences in the observed results between two versus three doses, since the 95% CI overlap. It is also possible that three doses are marginally more effective than two doses; however, these studies were insufficient in power to determine this. Based on these data and the results presented from the simulation (Table 2), it seems fairly obvious that in a given heartworm efficacy study, there is insufficient analytical power to say that one worm or zero worms in the treated group are different.

**Table 2:** Output of a simulation experiment based on an experiment where treated and non-treated control groups each with 10 dogs are inoculated with 50 L3 per dog, with a mean establishment rate of 50%. Simulation was repeated 1000X, and values represent the percent of times 0, 1, 2, or >2 worms were observed at five different efficacy levels

% of times you will see 0, 1, 2, or >2 worms				
Efficacy (%)	0 worms	1 worm	2 worms	>2 worms
99.95	88.16	11.07	0.74	0.03
99.5	28.39	35.61	22.45	13.55
99	8.32	20.74	24.90	46.04
98.5	2.6	8.68	16.09	72.63
98	0.72	3.04	8.59	87.65

## Methods and Results

### Statistical issues related to efficacy

Analysis of data to evaluate the treatment efficacy is challenging, especially when the number of animals involved in the study is small. Worm count data tend to possess a variety of complicating characteristics such as over-dispersion, asymmetric distribution, low counts, and excess zeros [9, 16]. These features in turn lead to high variability and hence, an accurate statistical assessment of efficacy is critical for a proper interpretation of the data. To statistically analyze such data, it is also important to identify sources of variability and develop statistical methods that take into account underlying biological issues.

As a first step towards describing the efficacy of a treatment, one needs to have a precise definition of efficacy. In this manuscript, we work with the following definition of efficacy (*e*):

1 $$ e=\frac{\mu_{ctl}-{\mu}_{tx}}{\mu_{ctl}} $$

Where *μ*_*ctl*_ is the population mean of worm counts in non-treated control dogs and *μ*_*tx*_ is the population mean of the worm counts in the drug-treated group. In the above formula, both *μ*_*ctl*_ and *μ*_*tx*_ are population level quantities and are unknown. Data obtained from a given biological experiment(s) are used to estimate the above parameters. Thus, while in experiments it may be the case that the estimates of *μ*_*tx*_ equal zero, yielding an observed efficacy of 100%, this may be not the case in many experiments if this same experiment was repeated multiple times. The frequency with which a given result is observed depends on the true efficacy of the drug at the tested dose, and the amount of variability in the experimental system. Data that shows as a function of efficacy, how often one would see zero, one, two, or more than two worms post treatment are shown in Table 2. From those data we notice that when the true efficacy is 99.95%, then 88% of the time we are unlikely to see any worms post treatment when experimental groups contain 10 dogs. Thus one obtains a false sense of 100% efficacy of a drug when the true efficacy is only 99.95%. In contrast, when the true efficacy of a drug is 99%, then only 8% of the time would we expect to observe 100% efficacy. Furthermore, when efficacy is 99% there is a high likelihood that we might see any one of the following results: zero, one, two or more than two worms (Table 2).

**Fig. 1:**
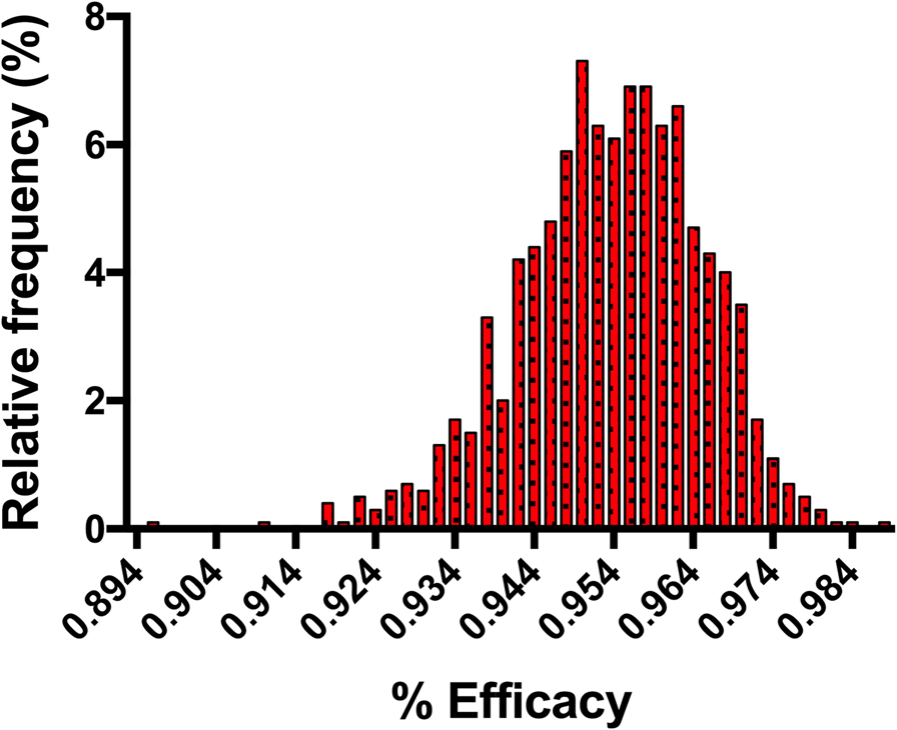
Efficacy distribution histogram of results from parametric bootstrap analysis of efficacy data for the milbemycin oxime group as reported in Blagburn et al. [17]. Mean and 95% confidence interval for efficacy are 95.3% (93.3, 97.1)

Returning to sources of variability, an additional issue arises, as it is impossible to determine what percentage of the inoculated iL3 remained viable in the treated group at the time the treatment was administered. Under the study design described above, the non-treated control animals are used to estimate the percentage of the inoculated iL3 remaining viable in the treated group at the time of treatment. This is referred to as the *establishment rate*. The establishment rate depends on an assortment of biological factors, including the dog’s immune system, and hence is likely to change among dogs in a given heartworm study, as well as between studies. In addition, the strain used in a given study is very likely to impact the establishment rate, although this has not been systematically examined. Thus, the estimate of the efficacy will vary between dogs and between studies and hence it is important to understand the estimated efficacy as a distribution rather than as a single number. Figure 1 illustrates this issue, showing a histogram of the efficacy determined when performing simulations using published data for milbemycin oxime [17]. Note that when a biological experiment is performed, we can only observe a single set of results. These results may be reflective of the true efficacy or may not be, depending on the amount of variability in the system and luck. By performing simulations (computer experiments) using the observed data and an appropriately designed analysis model, however, we can determine both the probability of a given result and the range of expected results. Because the simulation is based on the observed biological data of a single experiment, the average and median result of the simulation will be close to the observed result. One can see, however, that a rather wide range of observed efficacies are possible; in this case the reported efficacy was 95.3%, but one could have gotten results that ranged from 89% to 99% (Fig. 1).

Returning to Table 2, the data presented were generated in a simulation where the parasite establishment rate was assumed to have a mean of 50%, but allowing dog-to-dog establishment rate to vary. We selected 50% because it is close to the average seen across many heartworm studies in the published literature. Overall across published studies, mean establishment rates most commonly vary from approximately 30% to 70%, with individual dog establishment rates varying from 0 to 90%. Such large differences will greatly impact the probability of observing various results. Thus, as variability in establishment rates increases, and as true efficacy decreases (below the theoretical 100%), the amount of variability in the observed results will increase. Our simulation experiments show that a variability of just 5% in the establishment rate can introduce a variability of up to 13% in the observed efficacy. We also find that efficacy determinations that do not account for variability in establishment rate can over-estimate the efficacy in more than 40% of cases. Similarly, not accounting for between-dog variability can overestimate the true efficacy in 27% of the cases.

### Statistical modeling

We now describe the statistical model used for analyzing data from the study design described above. Let the number of dogs in the control group and treated group be denoted by n. Let N denote the number of worms inoculated into each dog, Y denote the number of worms recovered from a control dog, and X the number of worms recovered from a treated dog. These data when subscripted by k will denote the corresponding values for each dog. As an example, Y_k_ will denote the number of worms recovered from the k^th^ control dog while X_k_ will denote the number of worms recovered from the k^th^ treated dog. Because the establishment rate changes between dogs due to a variety of biological factors, we postulate that for the k^th^ dog

$$ {\mathrm{Y}}_{\mathrm{k}}\sim {\mathrm{g}}_{\mathrm{k}}(.),\kern0.5em {\mathrm{X}}_{\mathrm{k}}\sim {\mathrm{h}}_{\mathrm{k}}(.) $$

where g_k_(.) and h_k_(.) are probability distributions with means μ_c,k_ and μ_t,k_ respectively. The subscript k in the means indicate that these means can change between dogs, and because it is difficult to identify all the factors involved the term can be treated as being influenced by unobserved random variables. We call these *latent effects*. Some examples of these models are as follows:

1. Negative-Binomial /log normal model.
2. Poisson /log-normal model

The *population mean response*, which is the parameter of the worm count distribution, can also be allowed to vary between dogs. Specifically, one could model the worm count for a dog k in a treated or control group to be Poisson with mean λk, where λk changes smoothly according a distribution. This type of modeling is referred to as *hierarchical modeling* and the distribution describing the changes to λk is referred to as the *random effect* or *latent distribution*. It describes variability in the responses due to hidden or unobserved biological factors.

While these models are useful to account for different sources of potential variation, inferences based on these models are difficult. There are no closed form expressions for the means, and variances are typically obtained using numerical methods. The confidence intervals can be obtained from software such as SAS and R, but these analysis models are based on the assumption that the sample size is large. In our data sets, however, we have only 8 to 14 dogs per group, and this number cannot in general be considered as large.

An alternative approach to address the issue of small sample size is via the use of the bootstrap method [18] (Table 3). It is well known in the statistical literature that the parametric bootstrap method mimics the true inference (which would be the case when one knows the sampling distributions of the parameter estimates). For this reason, in the current paper we use the parametric bootstrap approach for inference concerning efficacies.

**Table 3:** Parametric bootstrap algorithm

Step 1	Denote by n_1_ and n_2_ the number of dogs in the control and treated groups in a study
Step 2	Use Information criteria to identify a statistical model for control data. The possible choices include negative binomial model, Poisson model, and their variants, which take into account unobserved variability using latent effect models.
Step 3	Using the fitted model obtain an estimate of the establishment rate and an estimate of the mean number of worms in the treated group.
Step 4	Using the above fitted model, and including the estimate of the establishment rate, and the mean number of worms remaining in the treated group, simulate the number of worms “available for treatment” and the number of worms remaining after treatment for each dog in the study.
Step 5	Using the result from Step 4, estimate the efficacy using the formula (1).
Step 6	Repeat Step 3 through Step 5 M times to obtain efficacies from M studies. This is typically done to yield results from 1000 or more studies.
Step 7	Order the efficacies from M studies from smallest to largest and obtain the confidence interval by taking the empirical quantile of levels α/2 and (1-α/2) as the lower and upper end-point of the confidence interval. Typical choices for α are 0.95 or 0.90

An important additional issue that arises in the data sets for heartworm studies is that the establishment rate is not observable. The bootstrap algorithm described here facilitates incorporating the estimated establishment rate and its variability in identifying the true efficacy. While parametric bootstrap is one approach, related Bayesian methods can also be applied to obtain similar results.

### Real-life data illustrations on the usefulness and power of statistics in addressing biological questions

#### Illustration 1: ZoeMo-2012 vs JYD-34

We performed the analysis described in Table 3 using SAS software (Version 9.4), on efficacy data for two strains of *D. immitis*, JYD-34 and ZoeMo, both of which are derived from the same source dog. In this analysis we used M = 1000 in the parametric bootstrap algorithm. Both JYD-34 and ZoeMo were established from blood samples collected from a heartworm microfilariae (MF) positive dog originally from Pittsfield, Illinois, but with little other known history. Thus the travel history, ML treatment history, and age of the dog, as well as the age of the heartworm infection are all unknown. JYD-34 was established first (by TRS Laboratories), as a blood sample was used to infect mosquitoes on 13 July 2010 and TRS recipient dogs were infected with 50 iL3s on 29 July 2010. The JYD-34 isolate was validated in April 2011 with dogs testing positive for both MF and adult heartworm antigen. The JYD-34 strain was later found to be resistant to ML drugs [19]. In contrast, the ZoeMo strain was established by Zoetis (Kalamazoo, MI) from a blood sample collected from the same source dog, but approximately 17 months later on 4 December 2012. On 19 December 2012, two dogs were each inoculated with 50 iL3 and these two dogs were positive for MF on 18 July 2013, validating passage of this strain. Interestingly, both of these strains were demonstrated to be ML-resistant, but the observed efficacies for the two strains were quite different [20]. This then begs the question: Are the differences in observed efficacy a result of random variability, or is there a real biological difference between the strains? To address this question we analyzed data from efficacy trials using both strains where a single 3.0 μg/kg oral dose of moxidectin was administered 30 days following inoculation with 50 iL3. Furthermore, we analyzed data from JYD-34 comparing efficacy results of a single 3.0 μg/kg oral dose of moxidectin administered on day 30 with three consecutive 3.0 μg/kg oral doses administered on days 30, 60, and 90.

##### ZoeMo-2012

The distribution of worm counts for the non-treated control and treated groups were determined to be Poisson based on Bayesian Information Criterion. Based on our analysis the 95% CI for the efficacy of moxidectin was (78.32, 84.38) with an average efficacy of 81.63. This analysis has the interpretation that for the observed number of worms in the moxidectin group, accounting for variability in the establishment rate, the interval (78.32, 84.38) captures the true efficacy 95% of the times. We refer to the above interval as a *conditional confidence interval*. On the other hand, if the set of dogs in the data are considered to be a random sample from a population of dogs, then the CI for the efficacy of moxidectin is (74.21, 88.08) with an average efficacy of 81.47. We refer to this confidence interval as a *marginal confidence interval.* From a regulatory perspective, we believe that the marginal CI is the more relevant value. Note that the average efficacy is virtually the same for both analyses, but taking all the sources of variability into account widens the CI.

##### JYD-34

We next turn to the JYD34 strain for a single dose of moxidectin. The distribution for the non-treated control and treated groups were determined to be Poisson based on the Bayesian Information Criterion. The conditional confidence interval for the efficacy was determined to be (7.05, 24.77) with a mean efficacy of 16.09%. The marginal 95% CI for a single dose of moxidectin for the JYD-34 strain was determined to be (2.02, 28.05) with an average efficacy of 15.91%.

We next examine multiple doses of moxidectin. The distribution for the non-treated control group was determined to be Poisson and the distribution for the treated group was determined to be negative binomial based on the Bayesian Information Criterion. The conditional 95% CI for the efficacy was determined to be (29.87, 43.99) with a mean efficacy of 37.38%. The marginal 95% CI for the efficacy was determined to be (19.95, 53.15) with an average efficacy of 37.65%.

#### Comparisons of the data

##### ZoeMo-2012 vs. JYD-34

To compare the efficacies of a single dose of moxidectin for ZoeMo-2012 and JYD-34, and a single versus multiple doses of moxidectin for JYD-34, we compared the distribution of the ratios of efficacies obtained using the parametric bootstrap model. If the differences in the observed efficacies were a result of randomness, then the 95% CI for the ratio of bootstrap efficacies should include 1.0. In both comparisons, however, this was not seen. The 95% CI for the ratio of bootstrap efficacies for a single dose of moxidectin for ZoeMo-2012 and JYD-34 was (0.05, 0.35). These results establish that moxidectin was significantly more efficacious for ZoeMo-2012 than it was for JYD-34.

##### JYD-34, single vs multiple doses of moxidectin

The 95% CI for the ratio of bootstrap efficacies of a single dose versus multiple doses of moxidectin was (0.09, 0.88) with an average of 0.46 That is, the average efficacy of multiple doses of moxidectin is approximately 2.17 times the average efficacy of the single dose of moxidectin. This result shows that multiple dose of moxidectin were significantly more efficacious than a single dose of moxidectin.

#### Interpretations of these analyses

##### ZoeMo-2012 vs. JYD-34

ZoeMo-2012 and JYD-34 are both derived from the same infected source dog, thus one would reasonably assume that these two strains would be very similar, and yield similar efficacy phenotypes. The only difference between them is that ZoeMo-2012 was established 17 months after JYD-34. In both cases, the strain was established using 50 iL3. Looking at the data from two experiments, performed using the same protocol at the same laboratory, it appeared that Zoe-Mo-2012 was less resistant than JYD-34. The eye test, however, is inadequate to make such a determination. Only by performing an appropriate analysis can we say whether those differences were most likely a result of random variability or of a biological cause. Here, we demonstrate that those results are indeed most likely due to a real biological difference. Several possible explanations exist, and one or more of these may be involved. The most likely explanation would seem to be that there was a mixed infection of both ML-susceptible and ML-resistant heartworms in the source dog, and that over the 17-month period there was a natural die-off of more resistant worms than susceptible worms, thus making the overall infra-population of worms infecting the source dog less resistant overall. If this is true it may be that the resistant worms were acquired prior to the susceptible worms and were simply dying of senescence preferentially due to their age. Alternatively, the resistant (or highly resistant) worms might have been less fit, and thus had a shorter life span than the susceptible (or less resistant) worms. An alternative explanation is that the genetic bottlenecks inherent in the passage and establishment of heartworm strains, by randomness, caused the newly established derived strains to be somewhat genetically different. These possible explanations are simply an effort to explain an observation, and may or may not be a correct interpretation. Based on the analyses reported here, however, one thing we can say with confidence is that there was a true biological difference in the infra-population of worms that became the strains JYD-34 and ZoeMo-2012.

##### JYD-34, single vs multiple doses of moxidectin

It is natural to expect that multiple doses of a drug are more effective than a single dose, and the observed efficacies in this case (37.7% vs 15.9%) lend credence to that expectation. Depending upon the number of dogs tested and the level of variability in the data, however, these differences may or may not be statistically significant. Here, our analysis demonstrates that this difference was not due to randomness, but rather to a real difference in the efficacy of the treatment regimens.

### Illustration 2: Efficacy of milbemycin oxime against the MP3 heartworm strain

#### Background to the issue

Two studies published in early 2011 received a great deal of attention, as these were the first reports in which less than 100% efficacy was seen with a single dose of an ML-containing heartworm preventive drug, when administered at doses previously demonstrated to be fully effective [10, 17]. Interestingly, although both studies demonstrated less than 100% efficacy, the efficacies they reported were vastly different. This was especially noteworthy at the time because these two studies were published in close time proximity to each other, the iL3 used for both studies were produced by the same lab (TRS Labs), under the same conditions, and the iL3 came from the same MP3 strain. Thus, one would expect there to be essentially no differences in the biology and genetics of the parasites used in the two studies. These disparate results led to a great deal of discussion and disagreement among the heartworm researcher community as to whether these differences were due to randomness or due to a real biological difference, and if biological, what the cause of the difference might be. The disparities in the results of these studies, and the disagreement this provoked among experts in the field, provide an excellent opportunity to illustrate how statistics can be used in a powerful way to gain important insights into biological questions.

#### Methods and results of the two studies

In Snyder et al. [10], three groups of 14 dogs were each infected with 50 MP3 strain *D. immitis* iL3 and then treated 30 days later with a label dose of either ivermectin or milbemycin oxime, or left untreated as controls. Five months post infection at necropsy, 13 of 14 dogs in the non-treated control group had adult heartworms, with a mean of 22.3 worms, whereas both the ivermectin and milbemycin oxime groups each had one dog with one worm, yielding a geometric mean efficacy of 99.8%. In Blagburn et al. [17], a similar but slightly different protocol was used. Here groups of eight dogs were used, but 100 rather than 50 MP3 strain iL3 were inoculated into each dog. Thus, the total numbers of iL3 administered per group of dogs were very similar for both studies (800 vs 700). Dogs were then treated 30 days later with a label dose of either ivermectin or milbemycin oxime, or left untreated as controls (note that selamectin and moxidectin groups were also included in that study, but this discussion will not address those data). In contrast to the results seen in the Snyder study, in the Blagburn study seven of eight treated dogs in both treated groups harbored worms at necropsy, with both the ivermectin and milbemycin oxime treated groups harboring a total of 23 worms each (range of 1–6 worms per dog). This yielded a geometric mean efficacy of 95.6%, and 95.4% for the ivermectin and milbemycin oxime treated groups, respectively. As a percentage of the number of iL3 inoculated into dogs in the two studies, the Blagburn study recovered 20 times as many adult worms at necropsy as the Snyder study. This begs the question: are the differences in the results of these studies compatible with an explanation of variability due to randomness, or is the magnitude of the difference better explained as being due to a biological cause?

#### Modeling of the data and results of parametric bootstrap analysis

**Fig. 2:**
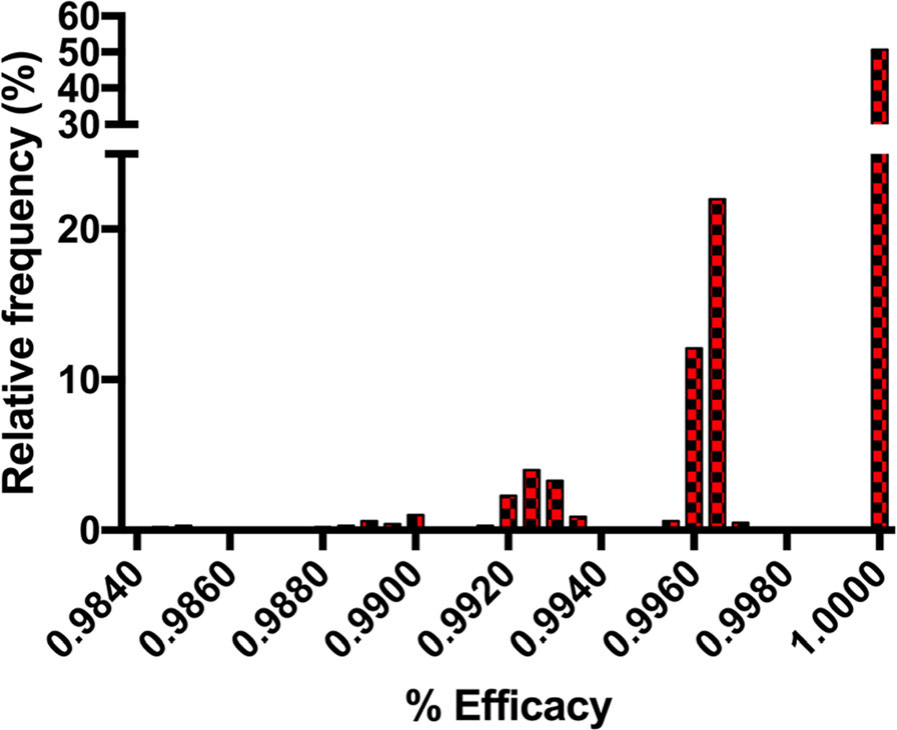
Efficacy distribution histogram of results from parametric bootstrap analysis of efficacy data for the milbemycin oxime group as reported in Snyder et al. [10]. Mean and 95% confidence interval for efficacy are 99.8% (99.2, 100). Note that although one worm was seen in the biological experiment, the parametric bootstrap analysis yielded 100% efficacy more than 50% of the time

To address this issue we analyzed the raw data for the milbemycin oxime group from both studies with the parametric bootstrap algorithm as described in Table 3 using SAS software (Version 9.4), with M = 1000. The distribution of worm counts for the non-treated control groups in both studies were determined to be Poisson based on Bayesian Information Criterion. Based on our analysis, the 95% CI for the efficacy of milbemycin oxime were (99.2, 100) and (93.3, 97.1), with average efficacies of 99.8%, and 95.3% for the Snyder and Blagburn studies, respectively. The distributions of the bootstrap efficacies are shown in Figs. 1 and 2. These figures reinforce the concept that if the same experiment is repeated many times (here M = 1000), different results would be seen very often. The frequency with which each result occurred in our analysis is illustrated in these figures. We note that the confidence intervals for efficacy for the two studies do not overlap, suggesting that the results are truly different. The most accurate way to address this, however, is not to compare the actual confidence intervals, but rather to compare the 95% CI for the ratio of bootstrap efficacies for the two studies. This analysis yielded an interval of (0.936, 0.974); since this interval does not include 1.0, we can conclude that the efficacy seen in the Snyder study was significantly greater than that seen in the Blagburn study. Furthermore, we can conclude that this difference is highly unlikely to have been caused by randomness, but rather must be due to a biological determinant. This analysis cannot determine what biological factors were responsible; the only means to determine this would be to develop hypotheses and then test those hypotheses in a biological system.

#### Interpretation and insights from these analyses

In the aftermath of these reports, leaders in the heartworm community presented many different hypotheses to explain these differences. Throughout those discussions, it was assumed that both studies had used the same heartworm strain, referred to as MP3. But did they really? Although these papers were accepted for publication within 2 months of each other, the studies were performed about 2 years apart; the Snyder study in 2008 and the Blagburn study in 2010. Neither publication provides details of the origin of the parasites used other than referring to them as from the MP3 strain. Recall from above that the MP3 strain was originally isolated and established in a dog at TRS Labs in 2006. The source of the MP3 parasites for the Snyder study was not the dog harboring the original infection, but rather from a dog that had been infected via transplant with 10 pairs of adult worms in 2008. In contrast, the iL3 used in the Blagburn study originated from a dog infected via inoculation of 50 iL3 about 2 years later (JW McCall, personal communication, 2017). Although details of how many passages occurred through different dogs are not available, it seems clear that at least several passages occurred via both adult worm transplant and infection with L3.

As discussed earlier, every time a heartworm strain is passaged a genetic bottleneck occurs, and with such small numbers of worms establishing on each passage, randomness can loom large. These two factors together, genetic bottlenecking and randomness, can have a major impact on the genetic diversity of the newly established version of the strain. To the recollection of the authors (RMK), this issue was never raised during the public discussions trying to find an explanation for the large differences between the studies. Rather, some proposed that randomness caused the parasites to differ in the two studies, and others looked for methodological differences. Given the results of our analyses for these two studies, however, and the analyses presented above for JYD-34 and ZoeMo-2012, it seems highly probable that a change in the genetics (genotype) of the MP3 ‘strain’ caused a corresponding change in the drug susceptibility phenotype of the MP3 ‘strain’ used in the second study. Although this may not explain the totality of the difference, it seems highly likely that it is the most prominent determinant. Although speculative, another possible contributing factor may have been the inoculation dose of iL3. As mentioned previously, the typical dose of iL3 administered in heartworm efficacy studies is 30 to 50 per dog, and our expectations of efficacy are based on this experimental model. But in the Blagburn study, 100 iL3 were used. If this was a contributing factor, it may suggest that efficacy decreases as parasite inoculum increases. This is an interesting question and deserves further investigation, particularly since recent studies suggest that the mechanism of action of ML drugs against *D. immitis* appears to involve the host immune response [21, 22].

## Discussion

In this paper we provide a detailed overview of the issues involved in the measurement of efficacy for heartworm drugs, and demonstrate that use of a hierarchical modeling approach with parametric bootstrap can provide elegant and statistical solutions to biological questions. Our results demonstrate how statistical modeling can improve the interpretation of data from heartworm efficacy studies by providing a means to identify the true efficacy range based on the observed data. Importantly, these new insights should help to inform regulators on how to move forward in establishing new statistically and scientifically valid requirements for efficacy in the registration of new heartworm preventative products. Furthermore, our results provide strong evidence that heartworm ‘strains’ can change their susceptibility phenotype over short periods of time, providing further evidence that a wide diversity of susceptibility phenotypes exist among naturally circulating biotypes of *D. immitis*.

For the first 20 years of testing ML-based heartworm preventives, all studies yielded 100% efficacy (at label doses), and this became the standard required by regulatory authorities for approval of new preventive products. Here, we make a case that insufficient numbers of heartworm strains/biotypes were used in testing to make such a conclusion, and we now know that “less susceptible” (MP3) and “resistant” biotypes (JYD-34) exist in the field, and are probably much more common than generally appreciated. Furthermore, our results demonstrate that even for what is considered a heartworm ‘strain,’ that the susceptibility phenotype can change over a short period of time, and this is likely due to changes in the genetic makeup of the strain that occur with each passage.

Given this reality, it has become necessary to reevaluate the 100% efficacy standard and even reevaluate what 100% really means. Results presented here demonstrate that using appropriate statistical modeling and simulation, we can begin to understand what it truly means when we observe 100% efficacy in a small heartworm efficacy trial, and likewise what it means when we see one or two worms. Our analyses also demonstrate that differences in establishment rates can greatly impact the measurement of efficacy, and we know from a multitude of studies that establishment rates vary widely both between dogs in a given study and between studies. Furthermore, the assumption of equal establishment rates in both the control and treated groups is inherent in the analysis and interpretation of all heartworm efficacy data published to date. Because of inherent variability, however, this assumption is certain to be untrue, and thus this effect must be taken into account when developing an appropriate analysis model.

We also provide two real-life illustrations where we used this statistical approach to address biological questions so that firm scientific conclusions could be drawn from the data, as opposed to having unsubstantiated and subjective opinion determine the interpretation. This same approach can be used on other data sets to address the core issues of the data, and can also be used to perform data simulations in order to better understand the issues at hand. Using this approach, it will be possible to develop new regulatory guidelines that are based on both sound data and sound statistical principles, and that permit the estimation of the interval that contains the “true” efficacy. Results of our analyses demonstrate that this is a superior approach to basing clinical and/or regulatory decisions on an “observed” efficacy, which may or may not closely mirror the true efficacy. Knowing that a diversity of susceptibility phenotypes exist among circulating biotypes of heartworm, such an approach will permit us to move forward based on scientifically sound information, and improve how we evaluate the efficacy of heartworm drugs.

We have demonstrated that observing 100% does not mean that a drug really is 100% efficacious against the strain it was tested against, let alone all circulating biotypes. Nevertheless, our simulation (based on 10 dogs, 50 iL3, and a 50% establishment rate) demonstrates that once efficacy falls below 99% it is likely that one or more worms will be seen, and once efficacy falls below 98% it is likely that two or more worms will be seen. Given these data, one could perform additional simulations and come up with a lower 95% CI that would be required to ensure that the true efficacy of a drug meets at a minimum, a very high standard, without requiring the 100% standard, which is not verifiable.

Finally, we find that most published heartworm efficacy studies do not provide the individual dog worm data, and only provide summary statistics for the entire group. This makes it impossible to re-analyze the data. Thus we feel it is imperative that in the future, all published heartworm efficacy studies include the individual worm data; reviewers should require this. In addition, publications in the heartworm literature do not provide the full history of the strain being used and only state the strain name, as if the strain is a biologically static entity. Although this is a vast improvement over the early heartworm literature that most often did not even mention the strain name, it is still not enough. Given the analyses presented here, it seems obvious that in every report or publication of a heartworm study that the full history of the strain used be provided in detail. Lastly, it would be ideal if each heartworm ‘strain’ used in registration trials was defined by a set of genotypic markers, such as microsatellites. This could then serve as a means to genetically fingerprint a strain, and potentially determine the degree of change occurring as it goes through successive passages.

## Conclusions

Powerful statistical models and data simulation provide a means to make accurate biological inferences on the observed efficacy. By linking biology and statistics, we are able to provide useful solutions to clinical questions, and improve our ability to make evidence-based decisions.

## Abbreviations

CI: Confidence interval
iL3: Infective third-stage larvae
MF: Microfilariae
ML: Macrocyclic lactones
